# Specialized Hidden Markov Model Databases for Microbial Genomics

**DOI:** 10.1002/cfg.280

**Published:** 2003-04

**Authors:** Martin Gollery

**Affiliations:** University of Nevada, Reno 1664 N. Virginia Street Reno NV 89557-0014 USA

## Abstract

As hidden Markov models (HMMs) become increasingly more important in the analysis of biological sequences, so too have databases of HMMs expanded in size, number and importance. While the standard paradigm a short while ago was the analysis of one or a few sequences at a time, it has now become standard procedure to submit an entire microbial genome. In the future, it will be common to submit large groups of completed genomes to run simultaneously against a dozen public databases and any number of internally developed targets. This paper looks at some of the readily available HMM (or HMM-like) algorithms and several publicly available HMM databases, and outlines methods by which the reader may develop custom HMM targets.


**Comparative and Functional Genomics**
*Comp Funct Genom* 2003; **4**: 250–254.
Published online 1 April 2003 in Wiley InterScience (www.interscience.wiley.com). **DOI:** 10.1002/cfg.280

**Conference Review**

### Specialized hidden Markov model databases for microbial genomics

Martin Gollery*
*University of Nevada, Reno, 1664 N. Virginia Street, Reno, NV 89557-0014, USA*

**Correspondence to: Martin Gollery, University of Nevada, Reno, 1664 N. Virginia Street, Reno, NV 89557-0014, USA. E-mail: mgollery@unr.edu*

Received: 27 January 2003
Revised: 5 February 2003
Accepted: 6 February 2003

### Abstract

**As hidden Markov models (HMMs) become increasingly more important in the analysis of biological sequences, so too have databases of HMMs expanded in size, number and importance. While the standard paradigm a short while ago was the analysis of one or a few sequences at a time, it has now become standard procedure to submit an entire microbial genome. In the future, it will be common to submit large groups of completed genomes to run simultaneously against a dozen public databases and any number of internally developed targets. This paper looks at some of the readily available HMM (or HMM-like) algorithms and several publicly available HMM databases, and outlines methods by which the reader may develop custom HMM targets. Copyright © 2003 John Wiley & Sons, Ltd.**

**Keywords:** HMM; Pfam; InterPro; SuperFamily; TLfam; COG; TIGRfams

### Introduction

Over the last few years, hidden Markov models (HMMs) have become one of the pre-eminent methods for the analysis of genomic data. Despite their slower search speeds, HMMs have gained in importance compared to other heuristic and dynamic methods, due to the ability of an HMM to represent an entire family of proteins in a single model. This enables the algorithm to weight the scoring as a function of the position, rather than a simple similarity score, which is the same for all locations, as in the Smith–Waterman or BLAST algorithms. As a result, it is possible for a BLAST search to show a high score and yet miss the residues that are most completely conserved in a family. Alternatively, it is possible for a BLAST hit to have a rather low score, yet demonstrate a match at all the crucial locations. Therefore, the scores (and therefore e-values) of a BLAST hit are no guarantee of a match.

This scenario is much less likely to occur with an HMM search. Since the score is penalized heavily if a highly conserved residue is not found, it is highly probable that a match with a high score will be a true homologue. As a result, HMMs have become very popular in the field of bioinformatics and a number of HMM databases have been developed. This paper reviews a number of these databases, with the goal of improving annotation through appropriate use.

### Types of HMM search programs

Several software packages have been developed for HMM analysis. These include:

- HMMER [3,4] (pronounced 'Hammer') was developed by Sean Eddy at Washington University and is the most popular implementation. The source code is freely available, as are executables for nearly all platforms, at **http://hmmer.wustl.edu/**
- SAM [8] was developed at UCSC, and is now on version 3.2 (**http://www.cse.ucsc.edu/research/compbio/sam**). A program called SAM-T02 allows you to search with a single sequence, and will then build a model from the resulting hits in a manner similar to PSI-BLAST. SAM uses a different file format than HMMER, but

Copyright © 2003 John Wiley & Sons, Ltd.

a conversion script has been written by Martin Madera and Julian Gough (**http://www.mrc-lmb.cam.ac.uk/genomes/julian/convert/convert.html**).

- Wise2 (**http://www.ebi.ac.uk/Wise2/**) was written by Ewan Birney, who is now at the EBI. Wise2 accepts HMMs in the HMMER format. GeneWise can search an HMM against EST sequences or genomic DNA, translating in all six reading frames and accounting for frame shifts and introns in the sequence. As a result of this extra sensitivity, GeneWise is quite slow.
- Meta-MEME [7] (**http://metameme.sdsc.edu/**) builds an HMM from motifs found with MEME, then trains it with data that you supply.
- HMMpro (**http://www.netid.com**) is a commercial package that extends the capabilities of the HMM to a more general model. HMMpro also features a nice viewer that makes it easier to interpret results. Single-user licences are free to academic users.
- PSI-BLAST [1] (**http://www.ncbi.nlm.nih.gov/blast**) builds a position-specific scoring matrix (PSSM) from the hits to a sequence that is submitted, and then compares that PSSM to the database in an iterated fashion, using any new hits to improve the model at each step.

### Available HMM databases

Selecting the appropriate target to search against is crucial to establishing the analysis pipeline. More than one target may be searched, although the speed will suffer as more searches are performed. If you find that speed is an issue, please refer to the 'speed solutions' section that follows.

Pfam [2] (**http://pfam.wustl.edu**) is the most well-known of the HMM databases. At the time of writing, PFAM includes over 5000 families. Pfam-A is hand-curated from multiple alignments/and is usually what is referred to simply as 'Pfam'. In addition, there are two versions of Pfam-A: Pfam-ls forces global alignments, i.e. the entire model must be matched. Pfam-fs is optimized for local alignments, so matches may include only part of the model. Pfam-B is automatically generated from ProDom, which in turn is generated from SP/TrEMBL.

The TIGRFAMs database (**http://www.tigr.org/tigrfams**) is hand-curated at The Institute for Genomic Research (TIGR). Information files containing protein family descriptions for use in annotation accompany each model. Information is given on 'equivalogs', which they define as, 'a set of homologous proteins that are conserved with respect to function since their last common ancestor'. TIGRFAMs consists of over 1600 models, as of version 2.1.

The SuperFamily database [6] (**http://www.supfam.org**) is maintained by Julian Gough at Stanford University/and Martin Madera at the MRC-LMB. Built from the SCOP (structural classification of proteins) database, which is built from PDB, SuperFamily provides structural (and implied functional) assignments to protein sequences at the superfamily level. One interesting feature of this database is that it is available in HMMER, SAM and PSI-BLAST formats.

The Simple Modular Architecture Research Tool [9] (SMART; **http://smart.embl-heidelberg.de**) provides over 600 hand-curated models in HMMER format, with particular emphasis on mobile eukaryotic domains. The SMART database is free to academic researchers but not to commercial users. Using the on-line website has several benefits over the local version, including queries by GO terms, links to OMIM and graphical output that is easily imported into publications. Use SMART if you are looking for signalling domains or extracellular domains.

NCBI's COG database (clusters of orthologous genes [12]; (**http://www.ncbi.nlm.nih.gov/cog/**), consists of over 3300 genes that are found in at least three lineages. The implication is that if this sequence is highly conserved across these lineages, then it must represent an ancient conserved domain. NCBI provides the COG database in several formats, including clustalW alignments. From these alignments, I have built HMMs to represent each of the COGs. The resulting database is currently in the testing stage, and is freely available on request.

HMMs reflect the data that they are trained on. Members of a protein family will have a different composition if they are found in an archaeon vs. a mammalian cell. Therefore, the TLFAM databases [5] were developed to provide more specific models for certain classes of organisms, e.g. TLFAM-Pro was trained exclusively on prokaryotic data and should theoretically provide optimal performance when used with new prokaryotic genomes. TLFAM-Pro has demonstrated higher scores and

Copyright © 2003 John Wiley & Sons, Ltd. 

longer alignments when analysing a new prokaryotic genome that was not used in the training set, but a search against PFAM yielded more total hits. Therefore, it is recommended that the TLFAM databases should be used as complements to PFAM, not as replacements.

Since TLFAM-Pro is based on prokaryotic data, it should yield poor results if used with a eukaryotic genome. Data from *Plasmodium falciparum*, an invertebrate eukaryote, was used as a negative control. As predicted, the average scores were lower and the average alignment lengths were shorter than those produced by PFAM.

TLFAM-Arc and TLFAM-Fun were produced from archaeal data and fungal data, respectively, and more databases are in production. However, a large amount of data has been entered into the public databases recently, so all of the TLFAM databases need to be revisited. The TLFAM-2 series is in development, and should be freely available by late spring of 2003. TLFAM-2 is being designed as a largely automated system and, as such, will include many more classes of organisms. One must consider, however, that as the dataset becomes more specific, there may not be enough sequences representing each protein to train the models. Interested parties may contact the author at: **mgollery@unr.edu.**

### Choosing a target

There is no single HMM database that will provide the best results for all data and all purposes. It is possible to search against all of these targets, if the time permits. Search time is a function of the total number and length of the models searched. For large amounts of data, the computational expense is simply too great. Moore's law has not helped, as the amount of data grows more quickly than the speed of the CPUs. The most common answer to this problem is to build a server cluster or computational grid. Another answer is to use software that is optimized for speed. Southwest Parallel Software (**http://www.spsoft.com**) has an optimized version of the HMMpfam program that is many times faster than the free version. This software runs on standard UNIX servers, and requires no alteration of existing scripts and protocols. This may be much less expensive than building and managing a cluster, or expanding an existing cluster. For those who require acceleration of hundreds to thousands of times faster than standard HMMER software, specialized hardware solutions are available from TimeLogic (**http://www.timelogic.com**) and Paracel (**http://www.paracel.com**).

Barring these solutions, one must consider 'target triage'. In order to pick the database that is most useful for the types of analyses that interest you, run a random subset of the data against all of the targets mentioned here. Only by careful checking of the output can you discover the most useful database for your particular needs. Over time, you should revisit this decision, as the data changes and new targets become available.

### Interpro

Submitting data to a number of databases spread across a number of servers and then compiling the results into some sort of understandable output can be a daunting task. Fortunately, the EBI has developed InterPro (**http://www.ebi.ac.uk/interpro/**). The InterPro database has over 7000 entries and is built from PFAM, Prints, Prosite, ProDom, SMART, TIGRFAMs and SP/TrEMBL. A program called InterProScan is available to be installed locally. InterProScan can automatically split large jobs into smaller chunks, submit them to different servers, reassemble the results, and assign InterPro numbers and GO mappings. It is scripted in PERL, so that researchers may easily modify the procedure to add new databases or new functionality. Due to the number of methods and databases that are used however, InterPro is quite slow. An informal test on a server farm with several hundred sequences of approximately 550 nucleotides each showed an average time of about 4 min/sequence per 2 GHz CPU. This throughput may be increased by one of the methods mentioned above.

### Custom databases

If the data of interest is not well represented in any of the existing databases, it is possible to custom-build models. Any dataset that lends itself to multiple sequence alignment will also be useful as an HMM. There are many ways to build a database of custom models, depending on the nature of the data and the goals of the

Copyright © 2003 John Wiley & Sons, Ltd. 

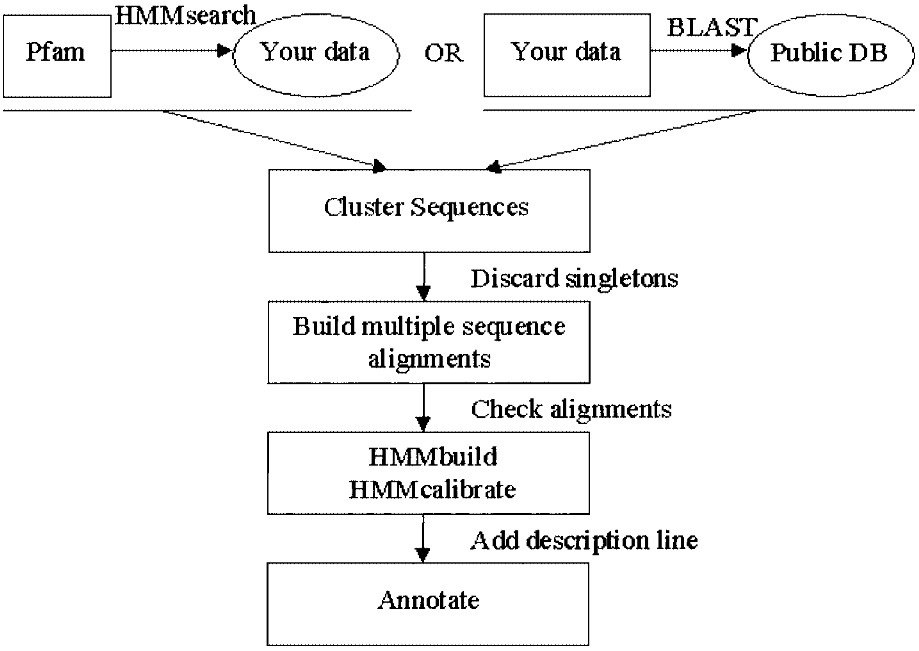

**Figure 1.** Building a database of custom HMMs project (Figure 1). The first step is to gather related protein sequences together, either through a BLAST search or by comparison to models in an existing database. These sequences are then aligned with ClustalW or some equivalent program. Singletons are discarded, and the alignments are inspected by hand to check for misalignments. The multiple sequence alignments (MSAs) are then built into HMMs with the program 'hmmbuild'. To set the scoring properly, the models must be calibrated using hmmcalibrate. This is a very slow process when used on a large group of models, and there are no accelerators to speed it up at this time. Once the models are built and calibrated, descriptions must be added to give an idea of what each model represents.

PSI-BLAST provides a method to produce a position-specific scoring matrix (PSSM) database that is quite similar in concept to an HMM database. The −C option will save the PSSM from a PSI-BLAST search. These matrices may then be processed with the 'Makemat' and 'Copymat' programs to prepare them for use with RPS-BLAST (reverse PSI-BLAST). This is the procedure used to produce the conserved domain database (CDD) at NCBI [10,11]. CDD contains over 10 000 PSSMs, representing data from SMART, Pfam and COG. Additional information on PSI-BLAST, RPS-BLAST and their options may be found in the readme files that come with the stand-alone BLAST distribution. RPS-BLAST ought to be investigated by all who are interested in developing their own databases, both for the relative simplicity to develop the files and for the speed of the resulting searches.

### Acknowledgements

I would like to thank John Cushman, Lee Weber and Gary Blomquist for their support, and Garrett Taylor for the programs that he wrote for the COG conversion. TimeLogic corporation supported the original version of the TLFAMs databases, and Gary Montry of Southwest Parallel Software provided terrific help and advice, along with his wonderfully fast executables.

### References

1. Altschul SF, Madden TL, Schäffer AA, *et al.* 1997. Gapped BLAST and PSI-BLAST: a new generation of protein database search programs. *Nucleic Acids Res* **25**: 3389–3402.
2. Bateman A, Birney E, Cerruti L, *et al.* 2002. *Nucleic Acids Res* **30**: 276–280.
3. Eddy SR. 2001. HMMER: profile hidden Markov models for biological sequence analysis (**http://hmmer.wustl.edu/**).

Copyright © 2003 John Wiley & Sons, Ltd. 

4. Eddy SR. 1998. Profile hidden Markov models. *Bioinformatics* **14**: 755–763.
5. Gollery M, Rector D, Lindelien J. 2002. TLFAM — a new set of protein family databases. *OMICS* **6**: 35–37.
6. Gough J, Karplus K, Hughey R, Chothia C. 2001. Assignment of homology to genome sequences using a library of hidden Markov models that represent all proteins of known structure. *J Mol Biol* **313**(4): 903–919.
7. Grundy WN, Bailey TL, Elkan CP, Baker ME. 1997. Meta-MEME: motif-based hidden Markov models of biological sequences. *Comput Appl Biosci* **13**(4): 397–406.
8. Krogh A, Brown M, Mian IS, Sjolander K, Haussler D. 1994. Hidden Markov models in computational biology: applications to protein modeling. *J Mol Biol* **235**: 1501–1531.
9. Letunic I, Goodstadt L, Dickens NJ, *et al.* 2002. Recent improvements to the SMART domain-based sequence annotation resource. *Nucleic Acids Res* **30**: 242–244.
10. Marchler-Bauer A, Anderson JB, DeWeese-Scott C, *et al.* 2003. CDD: a curated Entrez database of conserved domain alignments. *Nucleic Acids Res* **31**: 383–387.
11. Marchler-Bauer A, Panchenko AR, Shoemaker BA, *et al.* 2002. CDD: a database of conserved domain alignments with links to domain three-dimensional structure. *Nucleic Acids Res* **30**: 281–283.
12. Tatusov RL, Natale DA, Garkavtsev IV, *et al.* 2001. The COG database: new developments in phylogenetic classification of proteins from complete genomes. *Nucleic Acids Res* **29**: 22–28.

Copyright © 2003 John Wiley & Sons, Ltd. 
